# A neurocomputational account of reward and novelty processing and effects of psychostimulants in attention deficit hyperactivity disorder

**DOI:** 10.1093/brain/awy048

**Published:** 2018-03-13

**Authors:** Arjun Sethi, Valerie Voon, Hugo D Critchley, Mara Cercignani, Neil A Harrison

**Affiliations:** 1 Department of Neuroscience, Brighton and Sussex Medical School, University of Sussex, Brighton, UK; 2 Department of Psychiatry, University of Cambridge, Cambridge, UK; 3 Cambridgeshire and Peterborough NHS Foundation Trust, Cambridge, UK; 4 Sackler Centre for Consciousness Science, University of Sussex, Brighton, UK; 5 Sussex Partnership NHS Foundation Trust, Brighton, UK

**Keywords:** attention deficit hyperactivity disorder, methylphenidate, novelty, reward, substantia nigra

## Abstract

Computational models of reinforcement learning have helped dissect discrete components of reward-related function and characterize neurocognitive deficits in psychiatric illnesses. Stimulus novelty biases decision-making, even when unrelated to choice outcome, acting as if possessing intrinsic reward value to guide decisions toward uncertain options. Heightened novelty seeking is characteristic of attention deficit hyperactivity disorder, yet how this influences reward-related decision-making is computationally encoded, or is altered by stimulant medication, is currently uncertain. Here we used an established reinforcement-learning task to model effects of novelty on reward-related behaviour during functional MRI in 30 adults with attention deficit hyperactivity disorder and 30 age-, sex- and IQ-matched control subjects. Each participant was tested on two separate occasions, once ON and once OFF stimulant medication. OFF medication, patients with attention deficit hyperactivity disorder showed significantly impaired task performance (*P = *0.027), and greater selection of novel options (*P = *0.004). Moreover, persistence in selecting novel options predicted impaired task performance (*P = *0.025). These behavioural deficits were accompanied by a significantly lower learning rate (*P = *0.011) and heightened novelty signalling within the substantia nigra/ventral tegmental area (family-wise error corrected *P < *0.05). Compared to effects in controls, stimulant medication improved attention deficit hyperactivity disorder participants’ overall task performance (*P = *0.011), increased reward-learning rates (*P = *0.046) and enhanced their ability to differentiate optimal from non-optimal novel choices (*P = *0.032). It also reduced substantia nigra/ventral tegmental area responses to novelty. Preliminary cross-sectional evidence additionally suggested an association between long-term stimulant treatment and a reduction in the rewarding value of novelty. These data suggest that aberrant substantia nigra/ventral tegmental area novelty processing plays an important role in the suboptimal reward-related decision-making characteristic of attention deficit hyperactivity disorder. Compared to effects in controls, abnormalities in novelty processing and reward-related learning were improved by stimulant medication, suggesting that they may be disorder-specific targets for the pharmacological management of attention deficit hyperactivity disorder symptoms.

## Teaser

Novelty seeking is characteristic of ADHD, but how this influences reward-related decision-making is unknown. Sethi *et al.* show that stimulant medication selectively attenuates ADHD participants’ tendency to repeatedly select non-optimal novel options, and reduces substantia nigra novelty signalling. Stimulant medication increases learning rates and enhances performance in ADHD participants compared to effects in controls.

## Introduction

Attention deficit hyperactivity disorder (ADHD) is an early-onset neurodevelopmental disorder characterized by symptoms of inattention, impulsivity and/or hyperactivity with symptoms persisting into adulthood in up to 50% of patients (Simon *et al.*, 2009). Within the brain, both inattentive (Volkow *et al.*, 2007) and hyperactive/impulsive (Rosa Neto *et al.*, 2002) symptoms are linked to abnormalities in dopaminergic function, particularly within the mesolimbic reward system. Correspondingly, impaired reward learning (Frank *et al.*, 2007; Thoma *et al.*, 2015) has been theorized to play a central role in both the symptomatic expression and aetiology of this disorder (Luman *et al.*, 2010). Several theoretical approaches have emerged to describe how abnormalities in reward function are linked to the symptomatology of ADHD (Luman *et al.*, 2010). However, a framework that can explain these reward abnormalities across both neurobiological and neurocomputational levels remains to be articulated.

Over the past decade temporal difference and related Rescorla-Wagner learning models that allow computation of ‘hidden’ learning signals and quantification of learning from reward *in vivo* have provided a powerful method for characterizing human reward-related behaviour (Steinberg *et al.*, 2013). Through calculation of trial-by-trial prediction error signals these models have demonstrated a tight coupling between reward-related learning signals and dopaminergic neuronal activity (Schultz *et al.*, 1997; Hollerman and Schultz, 1998) within the substantia nigra/ventral tegmental area and ventral striatum (Montague *et al.*, 1996; Waelti *et al.*, 2001; McClure *et al.*, 2003; O'Doherty *et al.*, 2003; Bayer and Glimcher, 2005). This approach has helped clarify mechanisms of impaired reward-related processing in other disorders characterized by dopaminergic dysfunction including schizophrenia and Parkinson’s disease (Murray *et al.*, 2008; Rutledge *et al.*, 2009). More broadly, these models also present a theoretical framework for characterizing the behavioural impact of other salient influences, such as stimulus novelty, on decision-making processes and their instantiation within the brain (Wittmann *et al.*, 2008). Importantly, however, there is as yet no precise account of how reinforcement learning to reward is altered in ADHD, or how it is ameliorated by stimulant medication (Frank *et al.*, 2007; Luman *et al.*, 2010; Thoma *et al.*, 2015).

Preliminary work using computational modelling to simulate learning of reward, has indicated several candidate mechanisms that could account for ADHD-associated impulsivity (Williams and Dayan, 2005). For instance, reduced learning rates (slower updating of reward values with experience) are associated with reduced dopamine levels (Rutledge *et al.*, 2009), and may therefore mediate the association between impulsive reward-seeking and hypodopaminergia in ADHD (Williams and Dayan, 2005). Such an account may also help explain the efficacy of stimulant medication in improving reward-learning (Frank *et al.*, 2007; Thoma *et al.*, 2015), since dopaminergic medications enhance reward-related learning rates in Parkinson’s disease (Rutledge *et al.*, 2009).

In ADHD, aberrant novelty processing is a related, but critically underexplored, component of reward dysfunction. Stimulus novelty is a potent trigger for activation of dopaminergic neurons within the substantia nigra (Schultz, 1998); a mechanism that can bias preference towards novel options and drive exploratory behaviour (Kakade and Dayan, 2002; Wittmann *et al.*, 2008). Novelty preference is highly adaptive, enabling identification of new sources of potential reward and reducing uncertainty associated with unfamiliar stimuli. However, novelty preference also entails risk. Aberrantly high novelty valuation is linked to significant personal harm, including development of substance abuse (Wills *et al.*, 1994). It is therefore noteworthy that heightened novelty-seeking is robustly observed in ADHD (Downey *et al.*, 1997; Lynn *et al.*, 2005; Jacob *et al.*, 2014). Furthermore, novelty-seeking personality traits (Ebstein *et al.*, 1996; Ekelund *et al.*, 1999; Strobel *et al.*, 1999; Tomitaka *et al.*, 1999; Okuyama *et al.*, 2000; Kluger *et al.*, 2002; Munafo *et al.*, 2008; Roussos *et al.*, 2009) and ADHD (LaHoste *et al.*, 1996; Rowe *et al.*, 1998; Smalley *et al.*, 1998; Faraone *et al.*, 1999, 2001; Barr *et al.*, 2000; Eisenberg *et al.*, 2000) share genetic correlates in dopamine receptor (particularly DRD4) polymorphisms. To date, no work has specifically examined how increased novelty-seeking impacts reward learning in ADHD.

Reinforcement learning models may again help address this. Computational accounts of reward learning propose that novelty encourages exploratory behaviour through a fictive ‘bonus’ signal that enhances the reward value of novel stimuli (Kakade and Dayan, 2002). Supporting this, both novelty bonus and reward prediction error signals are associated with phasic dopaminergic activity in mesolimbic reward pathways (Steinfels *et al.*, 1983; Ljungberg *et al.*, 1992; Horvitz *et al.*, 1997; Kakade and Dayan, 2002; Bunzeck and Duzel, 2006; Wittmann *et al.*, 2008; Zald *et al.*, 2008; Schiemann *et al.*, 2012). Correspondingly, increased novelty bonus signals are observed in patients with impulse control disorders associated with Parkinson’s disease (Djamshidian *et al.*, 2011). We hypothesize that similar changes underpin impairments in impulse control characteristic of ADHD. Furthermore, it remains unclear why stimulant medications (that enhance synaptic dopamine) improve hyperactive/impulsive symptoms in ADHD, given the expectation that they would heighten novelty ‘bonus’ signals and potentially exacerbate these symptoms.

To address this, we tested 30 patients with ADHD and 30 matched control subjects on a reinforcement-learning task shown to be sensitive to effects of stimulus novelty on reward-related behaviour. Each participant completed the task during functional MRI on two separate occasions, once after taking stimulant medication and the other after placebo.

## Materials and methods

### Participants

Thirty adult ADHD patients were recruited from specialist clinics at Sussex Partnership NHS Foundation Trust. Assessment included semi-structured interview using the Diagnostic Interview for ADHD in Adults (DIVA), completion of the Conners’ ADHD self-report long version and Wender Utah questionnaires, informant history and wherever possible review of school reports. All had DSM-IV confirmed diagnoses of ADHD. Control participants were recruited through classified advertisements and university mailing lists. Participants gave written informed consent following full explanation of the experimental procedures. Local and national ethical approvals were obtained from Brighton and Sussex Medical School (14/014/HAR; 12/131/HAR) and the East of England (Hertfordshire) National Research Ethics Committee (reference: 12/EE/0256).

Exclusion criteria included past or current history of any neurological or psychiatric history, other than anxiety and/or unipolar depressive disorder currently in remission, past history of significant head injury, and current drug or alcohol abuse. Controls were additionally excluded if they had a history of serious cardiovascular conditions including cardiomyopathy, coronary artery disease, heart failure, ventricular arrhythmia or hypertension, current or recent use of monoamine oxidase inhibitors, coumarin anticoagulants, anticonvulsants or antipsychotics or a diagnosis of glaucoma. Of note, ADHD participants were routinely screened for these potential contra-indications to stimulant medication at clinical assessment.

ADHD and control participant were matched on age [mean ± standard deviation (SD) ADHD: 33.7 ± 9.51 years, controls: 32.6 ± 9.54 years, *F*(1,58) = 0.20, *P = *0.66], IQ [ADHD: 109.0 ± 6.57, controls: 110.1 ± 7.06, *F*(1,58) = 0.40, *P = *0.53], gender and handedness (Table 1). ADHD participants scored highly on both inattentive and hyperactive/impulsive domains. Each patient was managed on a stable regimen of methylphenidate (minimum 18 mg) or dexamphetamine (minimum 10 mg) for at least 2 months prior to study enrolment.

**Table 1:** Participant demographics and ADHD scores

Measure	Mean (SD)		F	*P*
	ADHD	Controls		
*n*	30	30	–	–
Male	19	19	–	–
Female	11	11	–	–
Age	33.7 (9.51)	32.6 (9.54)	0.2	0.66
Handedness			–	–
Right-dominant	28	29	–	–
Left-dominant	1	1	–	–
Ambidextrous	1	0	–	–
FSIQ^a^	109.0 (6.57)	110.1 (7.06)	0.4	0.53
CAARS ADHD Index	24.0 (5.30)	8.6 (5.01)	133.21	<0.001
Attention/memory problems	26.7 (5.46)	9.9 (5.67)	123.48	<0.001
Hyperactivity/motor restlessness	24.4 (6.46)	11.3 (5.68)	68.81	<0.001
Impulsivity/emotional lability	23.7 (7.36)	7.6 (4.12)	109.13	<0.001
Problems with self-concept	11.2 (4.72)	5.6 (4.45)	22.5	<0.001
DSM total ADHD score	37.6 (9.03)	12.8 (6.92)	159.66	<0.001
DSM Inattention	19.3 (4.46)	7.0 (4.55)	125.28	<0.001
DSM Hyperactivity and Impulsivity	18.3 (5.66)	5.7 (3.83)	110.44	<0.001

CAARS = Conners’ Adult ADHD Rating Scale; DSM = Diagnostic and Statistical Manual of Mental Disorders; FSIQ = Full scale intelligence quotient.

^a^As estimated by National Adult Reading Test (NART) scores.

### Experimental design

We used a randomized, repeated-measures, double-blind, placebo-controlled study design in which all participants attended two experimental sessions separated by a minimum of 1 week. Patients with ADHD were required to abstain from their regular ADHD medication for the test day and 2 days prior to testing. A.S., who performed all participant testing and processing of all behavioural and imaging data, was blind to treatment allocation. N.A.H. (a qualified doctor) was aware of treatment allocation for safety reasons but took no part in participant testing or data processing. At the start of the first session, all participants were randomized to receive either stimulant medication or placebo using sealed coded envelopes. The alternate treatment was given on the second experimental session. ADHD participants were administered either their normal morning dose of stimulant medication or an inactive placebo. Control participants received either 20 mg of methylphenidate or placebo.

### Reinforcement-learning task with novelty manipulation

**Figure 1:**
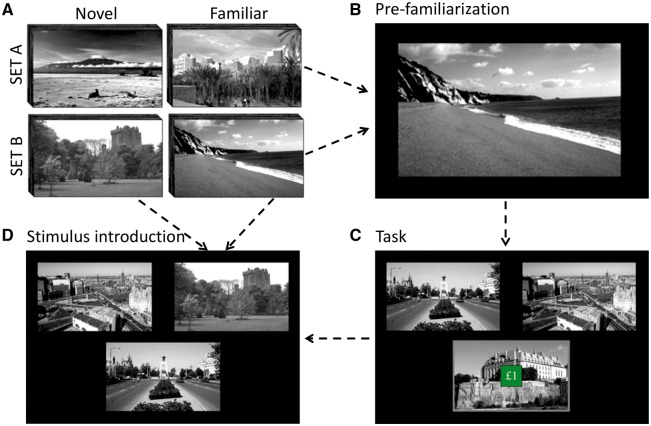
**Novelty processing task.** (**A**) Image sets: A set of 64 greyscale pictures (SET A or SET B) was randomly allocated for each session. (**B**) Pre-familiarization: participants were familiarized to half of the image set by passive, then active viewing. (**C**) Three-armed bandit task: during functional MRI, participants chose between three options (images) on each trial. Each option had a fixed probability (mean: 33%) of winning £1. Participants were instructed to choose options that maximized their total reward. Each trial consisted of stimulus presentation (3.5 s), choice feedback (3 s), and reward feedback (superimposed £1 or £0) (1.5 s). If participants failed to respond, ‘No response’ was displayed (4.5 s). There was a jittered intertrial interval (1.5–3 s). Option locations were randomly shuffled between trials. (**D**) On 25% of trials one option was randomly replaced by a new one from the image set. Images differed in reward value, but novel and familiar (pre-familiarized) images had the same reward probability distributions (mean 33%). The task was split into three 13-min runs, each containing 80 consecutive trials.

After drug administration, participants were immediately familiarized with 32 greyscale landscape images (Bunzeck and Duzel, 2006) over a 15-min session. This timing was important to ensure equivalent encoding (familiarization) across drug and placebo conditions. Ninety minutes after drug dosing, participants completed an MRI session (75-min duration), including three runs of the reinforcement-learning task (three-armed bandit task) encompassing a novelty manipulation (Wittmann *et al.*, 2008; Djamshidian *et al.*, 2011) (Fig. 1). Task structure followed Wittmann *et al.* (2008) to aid comparison with previously published findings. Task performance was timed to coincide with peak drug dopamine transporter occupancy (Volkow *et al.*, 1998).

### Computational modelling of choice behaviour

We characterized each participant’s trial-to-trial choices using the same model as Wittmann *et al.* (2008). This model included four free parameters: α learning rate, β inverse temperature or choice randomness, and Q_f_ and Q_n_, the initial values of familiar and novel stimuli, respectively. Initial values of each picture were set to Q_f_ if the picture had been pre-exposed during the familiarization phase, and Q_n_ if not. Values for the chosen option (*Q*) were updated according to the delta (*δ*) rule:

(1) Q(c,t+1)=Q(c,t)+α•δ(t)

Where *δ* denotes the reward (r) prediction error:

(2) δ(t)=r(t)−Q(c,t)

The probability of choosing an option was modelled according to a softmax selection strategy, where the probability of choosing an option *c* (out of the three options *k*) on trial *t* is:

(3) $$P(c,t)=\frac{exp(\beta •Q(c,t))}{\sum_{k=1}^{3}exp(\beta •Q\left(k,t\right))}$$

Model parameters were optimized on a per subject, per session basis using the interior point algorithm implemented in MATLAB’s fmincon function to minimize the negative log-likelihood of the observed sequence of choices. Model fit did not differ between ADHD and control groups. Novelty bonus was calculated as *Q_n_ − Q_f_*, with a positive value reflecting a preference for novel over familiar options.

To study effects of pharmacological manipulation and ADHD diagnosis on novelty processing we followed Wittmann’s approach and fit a second model where the initial value of novel and familiar stimuli were set equal i.e. *Q_n_* = *Q_f_* then compared the two models on the entire dataset (pooled over all subjects) using a likelihood ratio test. This model generated a second sequence of values *Q_base_*(*c*,*t*) and prediction errors *δ_base_*(*t*), representing baseline values without the additional effect of novelty. By comparing the two models, we calculated the additive value *Q_add_*(*c*,*t*)* = *(*Q*(*c*,*t*) − *Q_base_*(*c*,*t*)) and prediction error *δ_add_*(*t*) = *δ*(*c*,*t*)* − δ _base_*(*t*) associated with stimulus-novelty. Behavioural outcome measures included the four free model parameters, *α*, *β*, *Q_n_* and *Q_f_*. To study novelty specifically, we examined novelty bonus (*Q_n_* − *Q_f_*), tendency to pick novel options on their first presentation, and number of consecutive trials in which the novel object was selected.

Model-based regressors were generated for analysis of the neuroimaging data by entering each participant’s actual sequence of rewards and choices within the learning model to produce per-subject, per-trial estimates of the values *Q*(*c*,*t*) and error signals δ(*t*).

### MRI

T_2_*-weighted echo planar images were acquired on a 1.5 T Siemens Avanto equipped with a 32-channel head-coil using a −30° tilted acquisition to reduce orbitofrontal dropout (Deichmann *et al.*, 2003). Each volume provided whole brain coverage (34 interleaved ascending 3 mm axial slices, 1 mm interslice gap, echo time 43 ms: repetition time 2.52 s, in-plane resolution 3 mm). Multi-parameter mapping using three co-localized 3D multi-echo flash sequences was additionally acquired to provide magnetization transfer images with high contrast for our subcortical regions of interest (1.25 mm^3^ resolution, proton density: repetition time = 24 ms, echo time = 2.51– 21.9 ms, flip angle = 6°; T_1_: repetition time = 19 ms, echo time = 2.51–10.82 ms, flip angle = 20°; magnetization transfer: repetition time = 30 ms, echo time = 2.51–10.82 ms, flip angle = 12°) (Helms *et al.*, 2009; Sethi *et al.*, 2017). Magnetization transfer images were segmented then normalized in SPM8 (http://www.fil.ion.ucl.ac.uk/spm/) to aid group level anatomical localization. Diffusion weighted MRI and multi-echo resting state datasets were also acquired, though are not reported here.

Functional MRI data were analysed in an event-related manner in SPM8. Preprocessing consisted of spatial realignment, segmentation and normalization to a standard echo-planar imaging template then spatial smoothing with an 8 mm full-width at half maximum Gaussian kernel. Modelling of the data exactly replicated the approach used by Wittmann *et al.* (2008): each trial was modelled with impulse regressors at two time points: time of picture presentation (taken to be the time of decision), and time of outcome presentation (3 s after key press), *δ_base_*(*t*) and *δ_add_*(*t*) and Q-values (*Q_base_*(*c*,*t*) and *Q_add_*(*c*,*t*)), were used as parametric modulators of outcome and cue onsets, respectively. All regressors were convolved with a canonical haemodynamic response function and its temporal derivative. The six movement parameters were included as additional regressors to account for residual effects of scan-to-scan motion.

To enable inference at the group level, the coefficient estimates for the model-based regressor (*δ_base_*(*t*)) and the novelty bonus signal (*Q_add_*(*c*,*t*) and *δ_add_*(*t*)) from each individual subject and session were taken to allow second-level, random-effects group statistics to be computed in a mixed measures ANOVA [repeated factor: (drug, placebo), between-subject factor: group (ADHD, control)].

### *A priori* regions of interest

Bilateral ventral striatum and substantia nigra/ventral tegmental area were defined as *a priori* regions of interest, based on Wittmann *et al.*’s published findings. The ventral striatum region of interest was defined using the Martinez mask, which includes the nucleus accumbens and ventral caudate and putamen rostral to the anterior commissure (bilateral volume 5256 mm^3^) (Martinez *et al.*, 2003). Magnetization transfer images allow the substantia nigra to be easily distinguished from surrounding structures (Helms *et al.*, 2009; Sethi *et al.*, 2017). The substantia nigra/ventral tegmental area region of interest was therefore produced by manual tracing on the group mean magnetization transfer template produced using all participants’ normalized magnetization transfer saturation maps (bilateral volume 1792 mm^3^). Results are reported for clusters surviving a cluster forming threshold of *P < *0.001 and a stringent family-wise error (FWE) extent threshold of *P < *0.05 for the whole brain or appropriate region of interest.

### Questionnaires

The Conners self-report Adult ADHD Rating Scale (CAARS; Conners *et al.*, 1999) was used to index current ADHD symptom severity and the Tridimensional Personality Questionnaire (TPQ; Cloninger *et al.*, 1991) to measure trait novelty-seeking. The Beck Depression Inventory (BDI; Beck *et al.*, 1996) and State and Trait Anxiety Inventory (STAI; Spielberger, 1983) were used to assess depression and anxiety scores, respectively. The Multidimensional Personality Questionnaire (Tellegen and Waller, 2008) was also administered for use in a separate study. Behavioural analyses were performed in SPSS using mixed-measures ANOVAs followed by *post hoc t*-tests. Non-parametric Spearman’s rho was used to assess relationships between behavioural measures to account for non-normal distributions within the data.

### Behavioural analyses

Planned analyses assessed whether the model parameters *α*, β and total amount won on the task were affected by group or medication status using separate mixed-measures ANOVAs with group (between-subject) and medication (within-subject) factors. Planned analyses of effects of group and medication on novelty were assessed through inclusion of an additional within-subject novelty factor (*Q_n_*, *Q_f_*). To assess for long-term effects of medication on the behavioural novelty bonus, we also performed planned Spearman’s rho correlations between the novelty bonus and length of time on medication in months. Results of the above analyses were then further investigated using several *post hoc* behavioural tests as detailed in the relevant results sections.

## Results

### Sample characteristics

Mean (±SD) equivalent daily dose of methylphenidate was 50.0 ± 21.0 mg and mean psycho-stimulant treatment time 32.7 (±39.6) months for the ADHD group (see Supplementary material for further details). As anticipated, the ADHD group scored significantly higher on all CAARS subscales (Table 1). ADHD participants had significantly higher scores for depression and trait anxiety [BDI: ADHD = 13.7 (±8.6), controls = 5.6 (±6.6), *F*(1,58) = 17.01, *P < *0.001. STAI trait: ADHD = 53.5 ± 11.0, control s = 36.5 ± 10.7, *F*(1,58) = 36.51, *P < *0.001], though group differences in antidepressant use were not significant (ADHD = 6, controls = 1, Fischer’s exact test; *P = *0.10). BDI and STAI scores did not significantly correlate with variables of interest (task performance, novelty bonus, *α*, β; all *P* > 0.05).

Consistent with larger population studies (Downey *et al.*, 1997; Lynn *et al.*, 2005; Jacob *et al.*, 2014), ADHD participants scored significantly higher on novelty-seeking and harm-avoidance factors of the TPQ [novelty-seeking: ADHD = 22.9 ± 4.8, controls = 17.6 ± 5.6, *F*(1,57) = 15.29, *P < *0.001; harm-avoidance: ADHD = 17.1 ± 7.5, controls = 11.9 ± 7.5, *F*(1,57) = 15.29, *P = *0.01], but not reward-dependence [ADHD = 13.0 ± 4.0, controls = 12.3 ± 3.8, *F*(1,57) = 0.55, *P = *0.46] or persistence [ADHD = 5.3 ± 2.3, controls = 4.6 ± 2.0, *F*(1,56) = 1.38, *P = *0.245].

### Behavioural responses

OFF medication, ADHD patients showed impaired performance on the reinforcement-learning task (task performance) compared to controls {amount won [mean ± standard error (SE)]: ADHD: £86.3 ± 1.76, controls: £91.8 ± 1.80, *F*(1,58) = 5.17, *P = *0.027}. Moreover, stimulant medication had significantly different effects across the two groups (Group × Drug interaction), significantly enhancing ADHD participants’ performance compared to effects on controls who showed an inverse pattern of effects [change in amount won (mean ± SE): ADHD: £4.2 ± 2.26, controls: −£4.2 ± 2.22; *F*(1,58) = 6.95, *P = *0.011]. *Post hoc t*-test confirmed a trend towards increased performance following stimulant medication in the ADHD participants [*t*(29) = 1.84, *P = *0.077] and a converse trend towards decreased performance in controls [*t*(29) = −1.89, *P = *0.068].

To investigate these behavioural differences in more detail we then examined individual model parameters. Following Wittmann, we first tested whether the novelty bonus model better accounted for participants’ choices than the simpler model that initialized both sets of pictures with the best shared initial value (*Q_n_* = *Q_f_*), and found that it did (likelihood ratio test, 1 degree of freedom *P < *0.001). Consistent with what we observed for task performance, unmedicated patients with ADHD showed a significantly lower learning rate than unmedicated controls [*t*(58) = −2.34, *P = *0.011]. Stimulant medication also demonstrated dissociable effects on learning rates across groups [Group × Drug interaction *F*(1,58) = 4.17, *P = *0.046] significantly increasing learning rates in ADHD (mean ± SE, stimulant: 0.48 ± 0.06; placebo: 0.39 ± 0.04) compared to effects on controls (stimulant: 0.46 ± 0.06; placebo: 0.54 ± 0.05). However, though differences in effects of stimulant medication could be observed between groups, these did not survive within group comparisons (*post hoc t*-test *P > *0.1). Choice-randomness (*β*) did not significantly differ between groups or across medication condition (all *P > *0.1) (Table 2).

**Table 2:** Model parameter estimates in ADHD and controls

Measure	Mean scores (SE)	
	ADHD	Controls
**Placebo**		
Q_n_	0.57 (0.07)	0.46 (0.05)
Q_f_	0.52 (0.06)	0.45 (0.06)
*α*	0.39 (0.04)	0.54 (0.05)
*β*	7.58 (2.06)	8.69 (2.29)
**Drug**		
Q_n_	0.62 (0.06)	0.53 (0.05)
Q_f_	0.56 (0.06)	0.49 (0.05)
*α*	0.48 (0.06)	0.46 (0.06)
*β*	7.18 (1.54)	7.58 (1.27)

Across groups, unmedicated participants showed a strong preference for novel compared to familiar stimuli: [i.e. *Qn* > *Qf*; novelty bonus = £0.039 ± 0.01; *F*(1,58)_ = _10.84, *P < *0.005; see Table 2 for *Qf* and *Qn* for each condition and group separately]. Indeed, unmedicated ADHD participants expressed a novelty bonus more than double that observed in controls (£0.054 ± 0.018 versus £0.024 ± 0.015), though due to high interindividual variability this effect did not reach statistical significance [*F*(1,58) = 1.59, *P = *0.213].

To investigate whether group differences in novelty preference were expressed at the behavioural level, we then examined how frequently individuals selected novel compared to familiar stimuli on their first appearance. Unmedicated ADHD participants were significantly more likely than controls to choose novel compared to familiar options on their first presentation [Group × Familiarity: *F*(1,58) = 8.83, *P = *0.030] with *post hoc* analysis indicating a heightened salience of intrinsically ‘novel’ stimuli rather than an increased propensity to choose all newly introduced stimuli [% novel items selected: ADHD: 16.8 ± 1.23; control: 12.3 ± 1.09, *F*(1,58) = 8.83, *P = *0.004; % familiar items selected: ADHD: 15.3 ± 1.03; control: 14.0 ± 1.05; *F*(1,58) = 0.72, *P = *0.399].

### Relating novelty responses to drug-induced changes in task performance

We next investigated the relationship between novelty and task performance, by testing whether differences in ADHD participants’ responses to novel versus familiar stimuli underpinned interindividual differences in drug-related enhancement of performance on the task [(money won on stimulant − money won on placebo) / money won on placebo]. As anticipated, better performance ON medication was associated with a lower (i.e. more accurate) initial valuation of both novel (Qn) and familiar (Qf) stimuli (both rho = −0.53, *P = *0.009). However, persistence in selecting novel and familiar stimuli after their initial introduction differentially predicted task performance. Specifically, when ADHD participants were unmedicated, poorer performance was associated with greater persistence in selecting novel stimuli after their initial introduction (rho = −0.41, *P = *0.025) and a trend towards lower persistence in selecting familiar options (rho = 0.36, *P = *0.055). This persistence in selecting novel options in the drug-free state additionally predicted greater performance enhancement for ADHD participants when ON stimulant medication (*rho* = 0.46, *P = *0.011). No significant relationships were observed between novelty and drug-related changes in performance in the control group (*P > *0.1).

As the initial additive value of novel stimuli also decays as a product of learning rate, any increase in learning rate will result in a steeper decay of this value. Consequently, valuation biases of novel stimuli will reduce over fewer trials allowing potentially more accurate discrimination of high and low value novel options. To recap, we observed a significant increase in learning rate in ADHD participants compared to effects in controls following stimulant medication. To investigate whether this differential effect of medication on learning rate across groups improved choice behaviour towards novel stimuli we examined the number of consecutive trials in which participants chose novel options when they were actually the optimal choice (i.e. when the novel option actually had the greatest likelihood of a payoff of all available choices) or non-optimal (i.e. when the novel option did not have the greatest likelihood of a payoff of all available choices).

**Figure 2:**
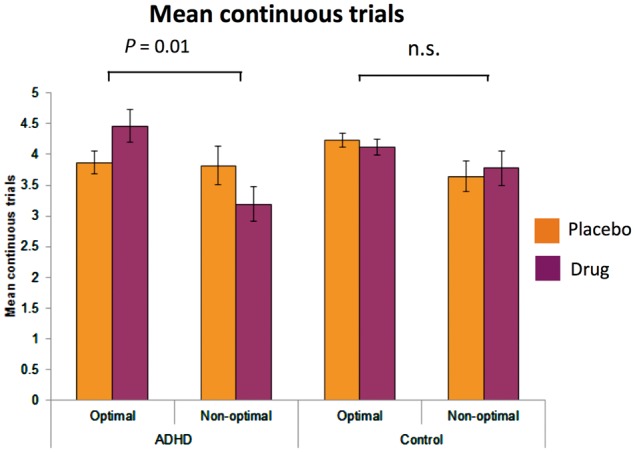
**Effects of stimulant medication on optimal versus non-optimal novel choices.** The mean number of times a novel option was continuously selected after introduction, separated according to whether it was the optimal choice (the highest value option of the three on screen) or non-optimal choice (not the highest value option of the three on screen).

Across groups, participants showed a greater tendency to persist with optimal rather than non-optimal novel options [*F*(1,58) = 10.04, *P = *0.002]. Neither medication nor group status showed main effects on this. However, medication did differentially affect how long the two groups selected optimal versus non-optimal novel options [Drug × Optimality × Group: *F*(1,58) = 4.80, *P = *0.032] (Fig. 2). Breaking this interaction down, in ADHD medication selectively enhanced persistence towards optimal compared to non-optimal novel options (Optimal: Drug: 4.47 ± 0.27; Placebo: 3.87 ± 0.19; Non-optimal: Drug: 3.20 ± 0.28; Placebo: 3.82 ± 0.31) [Drug × Optimality: *F*(1,28) = 7.60, *P = *0.010]. However, this pattern of effects was not observed in controls [Optimal: Drug: 4.12 ± 0.13; Placebo: 4.24 ± 0.11; Non-optimal: Drug: 3.77 ± 0.29; Placebo: 3.64 ± 0.25; Drug × Optimality *F*(1,28) = 0.25, *P = *0.624]. Across groups, participants also showed the expected tendency to persist with optimal rather than non-optimal familiar options [*F*(1,58) = 37.80, *P < *0.001]. However, medication did not differentially affect how long the two groups selected optimal versus non-optimal familiar options [Drug × Optimality × Group: *F*(1,58) = 0.49, *P = *0.485]. This indicates that stimulant medication selectively enhanced ADHD participants’ accuracy in discriminating optimal from non-optimal novel but not familiar options. This may reflect a steeper decay in the additive value of novelty induced by stimulant medication in the context of ADHD, which served to optimize decisions directed toward non-familiar (i.e. novel) stimuli.

### Effects of treatment duration on responses to novelty

Despite having a mean novelty bonus more than double that of controls, ADHD patients showed marked interindividual differences that overshadowed the statistical significance of group effects [*F*(1,58) = 1.59, *P = *0.213]. Previous studies show long-term alterations in striatal dopamine availability following sustained methylphenidate use (Wang *et al.*, 2013). We therefore investigated whether, individual differences in novelty bonus within the ADHD group related to duration of treatment. Strikingly, this analysis demonstrated a significant negative correlation between treatment duration and baseline (unmedicated) novelty bonus (rho = −0.44, *P = *0.018), i.e. patients treated the longest showed the lowest novelty bonuses.

### Relationship between reward- or novelty-related behaviour and clinical phenotype

Canonical correlation analysis was used to examine how reward and novelty related behavioural features [novelty bonus, learning rate, task performance (£won), and % novel options selected on first appearance, all on and off of medication] (set 1) related to clinical phenotype (inattention and hyperactivity/impulsivity domains of ADHD) (set 2). Briefly, this analysis derived latent canonical variates from linear combinations of each of the two sets of variables, to maximize their covariance. Results demonstrated a significant model [Wilk’s Lambda test: *F*(16,40) = 2.74, *P = *0.005] with a significant first (*P = *0.005) but not second (*P = *0.126) pair of canonical variates. The first novelty/reward derived canonical variate explained 63% of variance in the ADHD clinical phenotype canonical variate and 41% of overall ADHD phenotype (i.e. including items not captured in the ADHD canonical variate). The ADHD canonical variate was highly loaded by inattention scores (*r* = 0.99) and moderately loaded by hyperactivity/impulsivity scores (*r* = 0.54). The novelty/reward canonical variate was moderately loaded by OFF (*r* = −0.37) and ON medication learning rate (*r* = 0.36), ON medication amount won (£) (*r* = 0.53) and % novel items picked on first appearance (*r* = −0.65) and near-moderately loaded by OFF medication novelty bonus (*r* = 0.29) (all other variable contributions *r* < |0.3|). Overall, these data provide evidence that novelty/reward-related behavioural features explain up to 41% of the variance in clinical ADHD phenotype.

### Striatal and substantia nigra reward and novelty signals

**Figure 3:**
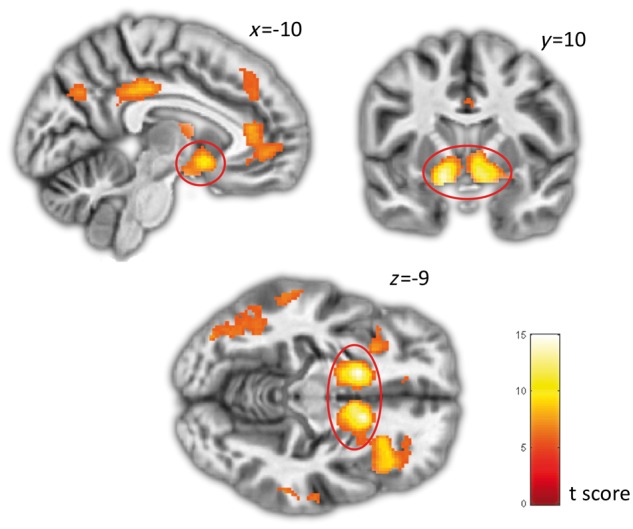
**Reward prediction error (*δ_base_*).** Brain regions significantly correlating with reward prediction error (*δ_base_*) across all participants and conditions. Peak activations in right and left ventral striatum are highlighted in red.

**Figure 4:**
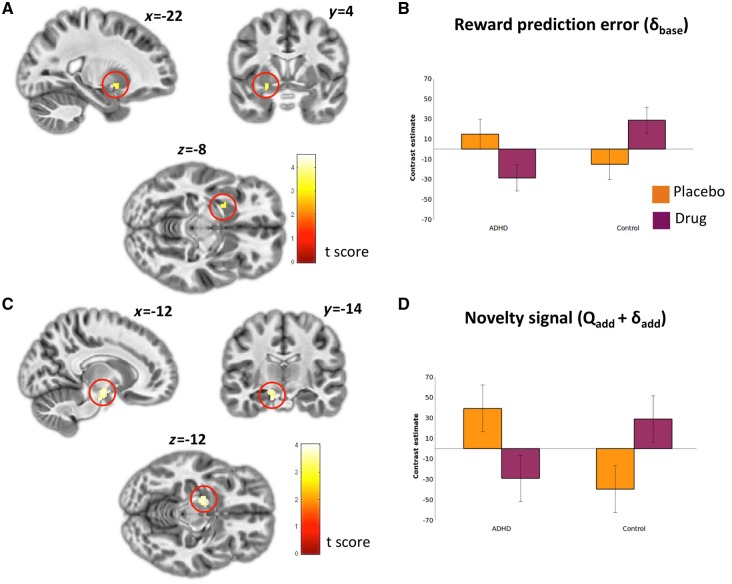
**Group by drug interaction for reward prediction error and novelty signals.** (**A**) Brain regions demonstrating a significant Group × Drug interaction for reward prediction error (*δ_base_*). Peak activation in the right ventral striatum highlighted in red*.* (**B**) Contrast estimate for right ventral striatum cluster. (**C**) Brain regions demonstrating a significant Group × Drug interaction for novelty signal (*Q_base_ + δ_base_*). Peak activation in the left substantia nigra highlighted in red*.* (**D**) Contrast estimate for left substantia nigra cluster.

Consistent with earlier reports (McClure *et al.*, 2003; O'Doherty *et al.*, 2003; Pessiglione *et al.*, 2006) computationally determined reward prediction error (δ_base_) showed a tight correlation (FWE: *P < *0.05) with bilateral ventral striatum and orbitofrontal cortex activity (and several other frontal and parietal regions) across groups (Fig. 3 and Supplementary Table 1). In addition, we observed a significant Group × Drug interaction for δ_base_ within the left ventral striatum [cluster SVC *P_FWE_* = 0.042; k = 15; *Z* = 3.51, coordinates = (−22 4 −8)], where the ADHD group exhibited a significant reduction in neural signals encoding reward prediction error while on stimulant medication compared to placebo. The opposite pattern was observed in controls (Fig. 4A and B).

Across conditions, we did not observe a corresponding correlation with novelty bonus signalling at the stringent thresholds used here. However, complementing our findings for reward prediction error, we observed a significant Group × Drug interaction in the substantia nigra/ventral tegmental area [whole brain cluster *P_FWE_* = 0.027, *k* = 107, *Z* = 3.67, coordinates = (−12 −8 −14)] indicating a significant reduction of novelty-related signalling in ADHD participants ON stimulant medication compared to placebo, and a converse pattern observed in controls (Fig. 4C and D). Corresponding to the reduction in behavioural novelty bonus observed in patients who had been ON medication for longer, activity within this cluster negatively correlated with time ON medication [small volume correction (SVC) FWE: *P = *0.003].

Finally, we sought to investigate whether drug-related reductions in baseline ventral striatal reward prediction error signalling or substantia nigra/ventral tegmental area novelty processing best explained the drug-induced enhancement of performance in ADHD. Consistant with our behavioural findings, drug-induced reductions in substantia nigra/ventral tegmental area novelty-bonus signalling (rho = −0.41, *P = *0.025), but not ventral striatal reward prediction error signalling (rho = 0.02, *P = *0.930) were related to improved task performance.

## Discussion

Our results provide evidence of impaired reward and novelty processing in ADHD and demonstrate attenuation of these deficits by stimulant medication compared to effects in controls. Furthermore, they identify a potential neurocomputational mechanism underpinning these abnormalities. Specifically, OFF medication ADHD participants displayed a greater tendency to choose novel (but not familiar) stimuli on their first presentation. This tendency was captured computationally as a higher (though statistically non-significant) novelty bonus and a significantly lower rate of value updating in response to reward (lower learning rate). This heightened salience of novelty, coupled with a slower decay in its rewarding properties, served to bias ADHD patients to repeatedly select novel options even when they were non-optimal. Interestingly, stimulant medication selectively remediated many of these abnormalities in ADHD compared to effects in controls. For example, compared to effects in controls, stimulant medication improved ADHD participants’ overall task performance, reduced their reward-learning rates and enhanced their ability to differentiate optimal from non-optimal novel choices. Aberrant persistence with selecting novel stimuli in the drug-free state additionally predicted ADHD participants’ response to treatment. These findings were complimented by our brain imaging data that showed that stimulant medication resulted in a significant reduction in substantia nigra/ventral tegmental area novelty-bonus signalling in ADHD participants, which significantly correlated with improved task performance. Finally, preliminary cross-sectional evidence suggested an association between long-term stimulant treatment and a reduction in the rewarding value of novelty. Together, these results highlight a central role for aberrant novelty processing in reward-related decision-making abnormalities observed in ADHD. Moreover, reward and novelty abnormalities appear to play an important role in the clinical phenotype of ADHD, with our canonical correlation analysis revealing up to 41% of the variance in clinical features of ADHD being explained by differences in reward and novelty processing.

Previous modelling with simulated data has predicted that hypo-dopaminergic abnormalities will reduce learning rates and in turn account for key components of impulsive reward dysfunction in ADHD (Williams and Dayan, 2005). By showing that patients with ADHD exhibit reduced reward-related learning rates OFF medication, we provide the first empirical evidence to support this. In addition, our data show a perturbation in the acquisition of reward-related behaviours in ADHD, supporting models that predict slower learning following positive reinforcement (Luman *et al.*, 2010). This reduction in reward-learning rate may also underlie observations of reduced adaptability to changing reward schedules (Kollins *et al.*, 1997) and increased temporal discounting (Williams and Dayan, 2005) and help explain why reward-related learning deficits appear more pronounced when rewards are probabilistic or intermittent rather than continuous (Aase and Sagvolden, 2006). Our results also suggest a tendency for stimulant medication to improve both learning rates and reward-learning task performance in ADHD participants compared to controls. However, it should be cautioned that though these differential effects were seen within groups in the raw behavioural data (e.g. persistence in selecting optimal versus non-optimal novel stimuli), effects on computational parameters (e.g. learning rate) only survived statistical thresholding in between group comparisons. Though supportive of differential effects of stimulant medications in ADHD patients and controls this will need to be confirmed in future large scale clinical studies.

At first glance, the increased novelty and reward prediction error signals we observed in ADHD may appear at odds with the hypo-dopaminergic profile believed to be central to this disorder. However, this divergence is predicted by a number of accounts of ADHD, which suggest that despite a reduction in tonic dopamine, phasic dopamine release is likely increased (Grace, 2001; Seeman and Madras, 2002; Cherkasova *et al.*, 2014; Badgaiyan *et al.*, 2015). While we cannot directly address this using our functional MRI data, we do show heightened error and novelty signals that are believed to be tightly linked to phasic dopamine (Schultz, 2007). One possible mechanism underpinning this heightened phasic novelty profile is lower mesolimbic D2/D3 receptor density in ADHD (Volkow *et al.*, 2009, 2011). Functionally, a reduction in D2/D3 receptors would lead to disinhibited phasic dopamine release (Volkow *et al.*, 2009, 2011), potentially explaining the increased sensitivity to stimulus novelty and persistence in selecting non-optimal novel options we observe. Evidence to support this comes from molecular imaging studies of trait novelty-seeking in the healthy population, where lower D2/D3 (auto)receptor binding in substantia nigra/ventral tegmental area is linked to higher novelty-seeking traits (Zald *et al.*, 2008). The reduction of substantia nigra/ventral tegmental area novelty-bonus signalling that we observe in ADHD participants after stimulant medication may equally reflect increased inhibition of these signals by D2/D3 activity, as methylphenidate exerts at least some of its therapeutic effects via increased dopamine binding to D2 receptors (Volkow *et al.*, 2012). Indeed, stimulant-induced enhancement of tonic dopamine is predicted to preferentially activate D2/D3 receptors that inhibit phasic dopamine (Dreyer *et al.*, 2010).

In contrast to the beneficial effects we observe in ADHD (reduced persistence in selecting non-optimal versus optimal novel stimuli), methylphenidate did not significantly affect behavioural responses to novelty in controls. Interestingly, previous work has shown that in other, broader cognitive domains, methylphenidate has similar effects in both ADHD patients and controls (Agay *et al.*, 2010). Thus, while stimulant medication appears to have equal impact on higher order cognitive functions in ADHD and controls (Agay *et al.*, 2010), it appears to engender different effects on processes related to reinforcement-learning to reward and novelty. Reinforcement-learning abnormalities may therefore reflect a disorder-specific therapeutic target for stimulant medication in ADHD. The fundamental origin of these differential effects remains unclear, though likely reflect baseline properties of the mesolimbic reward system. Indeed, while enhanced tonic dopamine and D2 activity may have a corrective role in ADHD and other hypo-dopaminergic disorders such as Parkinson’s disease (Rutledge *et al.*, 2009), increased D2 activity induced by methylphenidate in healthy controls (Volkow *et al.*, 2001) may explain their relatively poorer performance on drug compared to effects in ADHD. Correspondingly, selective D2 agonists appear to impair reward-learning in healthy subjects (Pizzagalli *et al.*, 2008).

A further, preliminary finding from our study was an association between long-term stimulant treatment and a relative attenuation of both novelty valuation and substantia nigra/ventral tegmental area responsiveness to novelty. The molecular mechanisms underpinning this potentially sustained improvement in novelty valuation are unclear. However, our findings may link observations that markers of ventral striatal D2/D3 reactivity predict long-term symptomatic improvements in attention (Volkow *et al.*, 2012), and prior associations between substantia nigra/ventral tegmental area D2/D3 receptor density and novelty-seeking behaviour (Zald *et al.*, 2008). Reductions in dopamine transporter (DAT) density after long-term methylphenidate treatment are largely interpreted as effects of tolerance (Wang *et al.*, 2013), yet a set of other neurobiological changes ascribed to methylphenidate use may also underpin potential long-term therapeutic benefits. These include increased neuroplasticity (Dommett *et al.*, 2008), dendritic spine formation (Kim *et al.*, 2009) and heightened expression of growth factors (Amiri *et al.*, 2013; Roeding *et al.*, 2014; Simchon-Tenenbaum *et al.*, 2015) within limbic circuitry supporting novelty processing, which may additionally contribute to long-term therapeutic effects, independent of current stimulant medication status.

In addition to effects on DAT, stimulant medications also block norepinephrine reuptake. Further, atomoxetine, a selective norepinephrine reuptake inhibitor with minimal action on DAT also shows clinical efficacy in ADHD (Del Campo *et al.*, 2011). Therefore, the differences in reward and novelty processing we observed may, at least in part, be mediated through noradrenergic mechanisms. This interpretation may also inform several discrepancies between our findings and other work. For instance, recent findings in monkeys using a highly selective DAT inhibitor have shown that though this increased the valuation of novel stimuli (broadly in line with our findings in healthy individuals), it elicited no change in learning rate (Costa *et al.*, 2014). This suggests that our observed effects on learning rate may have been mediated through noradrenergic mechanisms. This interpretation may similarly apply to our association between long-term medication use and a lower novelty bonus as individuals with Parkinson’s disease and compulsive behaviours arising from dopamine agonist exposure are reported to show elevated novelty preference (Djamshidian *et al.*, 2011). Alternately, this discrepancy may reflect different dopaminergic mechanisms of action (e.g. agonists, versus dopamine reuptake inhibitors) or different baseline properties of the dopamine system in these populations. With regards the latter point, it is noteworthy that short-term effects of these medications appeared to differ according to the population studied (in this case ADHD versus healthy controls). Further, not all individuals with Parkinson’s disease given dopaminergic medications develop impulsive and compulsive behaviours. While our current work cannot ultimately disentangle differential dopaminergic and noradrenergic effects, future work may seek to examine the effects of selective noradrenergic re-uptake inhibitors such as atomoxetine on novelty signalling in ADHD and control participants.

This work must be interpreted in the light of several limitations. First, we report results from a sample with prior exposure to stimulant medication. Though this was done to ensure a sample in which these medications were clinically efficacious, differential effects of medication observed across groups may have been influenced by differences in prior exposure. Second, our task was designed to mirror that of Wittmann *et al.* (2008) in healthy controls. A weakness of this task design is that it does not allow complete temporal separation of Q and δ [correlation of Q and δ across trials suggested 18.4% of shared variance (R^2^)]. Though this indicates that our prediction error results are unlikely to be substantially contaminated by differences in value signals, it did mandate that like Wittmann, our primary outcome variable (novelty bonus) was a combination of Q_add_ and δ_add_. Finally, our data were acquired at a field strength of 1.5 T. Though signal-to-noise will have been enhanced by our use of a 32-channel head coil, a higher performing system may have been sensitive to smaller effects in regions we have not reported.

Further work is required to consolidate the broader clinical implications of the aberrant responses to novelty observed here. For example, in addition to apparent roles in inattention and poor decision-making, heightened novelty valuation could well contribute to the high prevalence of substance use disorders observed in ADHD. Conversely, the apparent reduction in novelty valuation we observe with prolonged treatment could underlie the reported reduction in substance abuse risks associated with long-term medication use (Wilens *et al.*, 2003; Mannuzza *et al.*, 2008). Longitudinal data are clearly required to investigate this hypothesis. To conclude, our findings suggest that novelty valuation has an important role in defining the ADHD phenotype and likely treatment response. Indeed, effects of methylphenidate on novelty processing revealed a remarkably disorder-specific effect not observed for other broader neuropsychological domains (Agay *et al.*, 2010). Thus, while some of the beneficial effects conferred by stimulant medication appear compensatory rather than corrective, actions on reinforcement-learning, and novelty processing in particular, appear to represent specific pathological targets.

## Supplementary Material

Supplementary Data

## Abbreviation

ADHD: attention deficit hyperactivity disorder
