# Supplementary material for: A neurocomputational account of reward and novelty processing and effects of psychostimulants in attention deficit hyperactivity disorder
Source: Brain. 2018 Mar 13;141(5):1545–57. doi: 10.1093/brain/awy048 (PMC5917772; doi:10.1093/brain/awy048)
Supplement: Supplementary Data [file awy048_brain-2017-01636-file006.pdf]

SUPPLEMENTARY INFORMATION

RESULTS

Medication

Twenty-eight ADHD participants were treated with methylphenidate and two with dexamphetamine. Within those taking methylphenidate, a variety of different regimens were observed (Supplentary Figure 1). Several ADHD and control participants were also taking selective-serotonin reuptake inhibitor (SSRI) or selective-noradrenaline reuptake inhibitors (SNRI) antidepressant. Fischer's Exact Test (FET) detected no significant different between the groups (ADHD = 6, Controls = 1, p = .103).

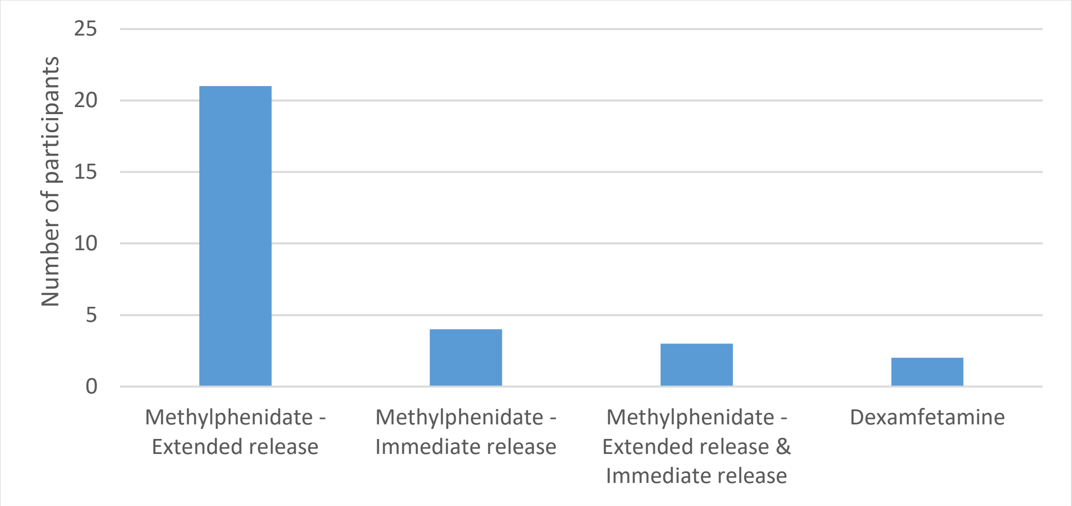

Supplementary Figure 1. Distribution of medication regimens in the ADHD group

In order to calculate approximate equivalent doses between methylphenidate and dexamfetamine, dexamfetamine doses were doubled. This is supported by various studies performing comparative analysis of the two drugs (for a review, see (1)), as well as the maximum recommended dose according to NICE guidelines. The mean methylphenidate dose in mg for the ADHD group was 50 ± 21.0. Excluding participants currently taking dexamfetamine, daily methylphenidate dose in mg was 49.7 ± 21.7.

Immediate release methylphenidate dose for controls was matched to the daily doses observed in the ADHD group. The equivalent immediate release dose for the ADHD group was therefore calculated based on mean daily dose, to account for differences in drug formulation and release schedule. The NICE recommendation of an immediate-release methylphenidate dose schedule of 1-4 times (mean 2.5), therefore indicates a 20mg equivalent acute dose. As such, 20mg of immediate-release methylphenidate was administered to controls. All medication was concealed in an opaque, easily absorbed capsule to ensure both participant and researcher (AS) were blind to treatment. A consultant psychiatrist (NAH) was aware of treatment allocation to ensure safety, though played no role in face-to-face participant testing or blinded pre-processing of neuroimaging data.

Supplementary Table 1: Correlations with reward prediction error (δ_{base})

Side	Region	Peak coordinates	*Z*	*K* (cluster)	*FWE p* (ROI)
L	Ventral striatum	[-12 8 -12]	>8	539	<0.001
R	Ventral striatum	[14 12 -10]	>8	733	<0.001
L	SN/VTA	[-8 -24 -14]	3.29	2	(0.02)
R	SN/VTA	[8 -14 -12]	3.56	13	(0.007)
L	Inferior frontal, orbitalis	[-30 22 6]	6.71	186	<0.001
R	Inferior frontal, orbitalis	[35 25 -10]	7.30	570	<0.001
L/R	Posterior cingulate	[0 -28 38]	>8	1333	<0.001
L	Inferior parietal	[-54 44 48]	>8	1845	<0.001
R	Inferior parietal	[52 -56 32]	6.46	473	<0.001
L/R	Medial prefrontal	[4 40 16]	7.37	1249	<0.001
L	Middle temporal	[-58 -42 0]	7.29	832	<0.001
R	Middle temporal	[62 -38 -2]	6.63	274	<0.001
L	Superior frontal	[12 46 46]	6.75	219	<0.001
R	Superior frontal	[-14 36 48]	6.94	552	<0.001
L	Insula	[-38 0 6]	5.55	34	<0.001
R	Insula	[40 2 4]	6.62	90	<0.001

R	Cerebellum	[36 -70 -38]	6.48	83	<0.001
L	Precuneus	[-4 -68 34]	6.25	83	<0.001
L	Pars Orbitalis/triangularis	[-48 38 0]	6.12	211	<0.001
L	Pars triangularis	[-52 12 14]	6.00	83	<0.001
L/R	Thalamus	[8 -2 14]	5.87	219	<0.001
L	Middle frontal	[-40 12 44]	5.83	72	<0.001
R	Pars triangularis	[54 24 16]	5.30	33	<0.001

SI REFERENCES

1. Arnold, L. E. Methyiphenidate vs. amphetamine: Comparative review. *J. Atten. Disord.* **3,** 200–211 (2000).
